# Serum levels of sclerostin as a potential biomarker in central arterial stiffness among hypertensive patients

**DOI:** 10.1186/s12872-018-0955-5

**Published:** 2018-11-27

**Authors:** Yu-Chi Chang, Bang-Gee Hsu, Hung-Hsiang Liou, Chung-Jen Lee, Ji-Hung Wang

**Affiliations:** 1 School of Medicine, Tzu Chi University, Hualien, Taiwan; 2 Division of Nephrology, Buddhist Tzu Chi General Hospital, Hualien, Taiwan; 3 Division of Nephrology, Hsin-Jen Hospital, New Taipei City, Taiwan; 4 Department of Nursing, Tzu Chi College of Technology, Hualien, Taiwan; 5 Division of Cardiology, Buddhist Tzu Chi General Hospital, No. 707, Section 3, Chung-Yang Road, Hualien, 97004 Taiwan

**Keywords:** Arterial stiffness, Carotid–femoral pulse wave velocity, Hypertension, Sclerostin, Dickkopf-1

## Abstract

**Background:**

Sclerostin is known to be a canonical Wnt/β-catenin signaling pathway inhibitor, while the Wnt/β-catenin signaling pathway is proposed to be involved in the development of arterial stiffness. This study aims to investigate the relationship between serum sclerostin levels and carotid–femoral pulse wave velocity (cfPWV) among hypertensive patients.

**Methods:**

Fasting blood samples were obtained from 105 hypertensive patients. Patients with cfPWV values of > 10 m/s were classified in the high arterial stiffness group, whereas those with cfPWV values of ≤10 m/s were assigned to the low arterial stiffness group. Serum sclerostin and Dickkopf-1 (DKK1) levels were quantified using commercially available enzyme-linked immunosorbent assays.

**Results:**

Thirty-six hypertensive patients (34.3%) who belonged to the high arterial stiffness group were generally older (*p* < 0.001), presented with lower estimated glomerular filtration rates (eGFR, *p* = 0.014), higher incidence of diabetes mellitus (*p* = 0.030), average systolic blood pressures (SBP, *p* = 0.013), pulse pressure (*p* = 0.026), serum creatinine levels (*p* = 0.013), intact parathyroid hormone levels (iPTH, *p* = 0.003), and sclerostin levels (*p* < 0.001) than their counterparts in the low arterial stiffness group. A multivariable logistic regression analysis identified sclerostin as an independent predictor of arterial stiffness in hypertensive patients (odds ratio, 1.042; 95% confidence interval (CI), 1.017–1.068; *p* = 0.001). Multivariable forward stepwise linear regression analysis also showed that serum sclerostin level (β = 0.255, adjusted R^2^ change: 0.146, *p* = 0.003) was positively associated with cfPWV values in patients with hypertension.

**Conclusions:**

In this study, serum sclerostin level, but not DKK1, is found to be positively correlated with cfPWV values and is identified as an independent predictor of arterial stiffness in hypertensive patients after adjusting for significant confounders.

## Background

Hypertension is a primary risk factor for morbidity and mortality, which leads to the development of atherosclerosis and target organ damage such as cerebral vascular events, retinopathy, and heart failure [1]. Several studies have reported arterial stiffness to be closely related to cardiovascular morbidity and mortality in hypertensive patients [2–4]. These evidences seem to point towards the hypothesis that central arterial stiffness is in fact a precursor rather than a sequela of hypertension [5].

Arterial stiffness, which results from progressive breakdown of elastic fibers in the aorta and large elastic arteries, causes increased pulse wave velocity (PWV), decreased diastolic coronary perfusion pressure, and will eventually lead to diminished myocardial oxygen delivery [6, 7]. Carotid–femoral PWV (cfPWV), calculated as the time required for arterial pulse to spread from the carotid to the femoral artery, can be used to measure central arterial stiffness [8]. Today, it is not only the gold standard to assess central arterial stiffness but also an independent predictor of the outcome of well-recognized cardiovascular events [3, 9]. Moreover, cfPWV has been recommended as a tool for the diagnosis of target organ damage in patients with earlier stages of hypertension [10].

When a Wnt ligand binds with its low-density lipoprotein receptor-related protein (LRP5/6) and Frizzled co-receptors on the cell surface of osteoblasts, intracellular β-catenin degradation is decreased, and this β-catenin then triggers targeted gene transcription to regulate bone turnover [11]. High-phosphate environment activates the Wnt/β-catenin signaling pathway and promotes osteogenic transdifferentiation and calcification of vascular smooth muscle cells through direct modulation of Runx2 gene expression [12]. The Wnt/β-catenin pathway mediates oxidized low-density lipoprotein-induced endothelial injury via oxidative stress and it plays an important role in the procession and development of atherosclerosis [13]. Another study noted that the Wnt/β-catenin signaling pathway is the master upstream regulator which controls the expression of multiple intrarenal renin-angiotensin system genes and plays an essential role in the pathogenesis of hypertension [14]. Thus, we can see that the Wnt/β-catenin signaling pathway contributes to the development of vascular calcification, atherosclerosis and hypertension. Sclerostin and Dickkopf-1 (DKK1) interact with the extracellular domains on LRP5 and LRP6 to competitively prevent the binding of various Wnt ligands to these coreceptors and is a typical Wnt/β-catenin signaling pathway inhibitor [15, 16]. More recently, studies showed that the expression of Wnt/β-catenin signaling inhibitors such as sclerostin and DKK1 may avoid further vascular calcification [17, 18]. However, the relationship between central arterial stiffness and serum levels of sclerostin and DKK1 among hypertensive patients remains unclear. In this study, we aimed to investigate this association in patients with hypertension.

## Methods

### Patients

Between January 2012 and April 2012, 115 hypertensive patients were recruited in the cardiovascular outpatient department (Dr. Ji-Hung Wang) of a medical center located in Hualien, Taiwan. Among the initial 115 hypertensive participants, 10 were excluded on account of acute infection (*n* = 1), acute myocardial infarction (*n* = 1), arrhythmia (*n* = 1), pulmonary edema (*n* = 1), or refusal to provide informed consents (*n* = 6). In the end, a total of 105 hypertensive participants, 69 men and 36 women, were included in the study. The Protection of the Human Subjects Institutional Review Board of Tzu Chi University and Hospital approved this study. All participants provided their written informed consents to take part in this study. Trained staff measured the morning blood pressures (BP) of our participants who have rested for at least 10 min using standard mercury sphygmomanometers with appropriate cuff sizes. Systolic BP (SBP) and diastolic BP (DBP) values were recorded at the points of appearance and disappearance of the Korotkoff sounds, respectively. SBP and DBP values were measured three times at 5-min intervals and were averaged for the analysis. For the prevalence survey, hypertension was defined as SBP ≥ 140 mmHg and/or DBP ≥ 90 mmHg, or patients who were taking antihypertensive drugs. Diabetes mellitus (DM) were diagnosed as patients whose fasting plasma glucose levels ≥126 mg/dL (6.1 mmol/L) or those who were using hypoglycemic agents [19].

### Anthropometric measurements

We measured the body weight of our patients wearing light clothing and without shoes, rounded to the nearest 0.5 kg; and height to the nearest 0.5 cm. We calculated the body mass indexes using the formulae of the weight in kilograms divided by the height in squared meters [20–22].

### Biochemical investigations

Fasting blood samples (approximately 5 mL) were immediately centrifuged at 3000 *g* for 10 min. Serum levels of blood urea nitrogen (BUN), creatinine, fasting glucose, total cholesterol (TCH), triglycerides (TG), high-density lipoprotein cholesterol (HDL-C), low-density lipoprotein cholesterol (LDL-C), total calcium, and phosphorus were measured using an autoanalyzer (COBAS Integra 800, Roche Diagnostics, Basel, Switzerland) [20–22]. We used commercially available enzyme-linked immunosorbent assays to measure the levels of serum sclerostin, DKK1 (Biomedica Immunoassays, Vienna, Austria) and intact parathyroid hormone (iPTH, Diagnostic Systems Laboratories, Texas, USA) [20]. We also calculated the estimated glomerular filtration rate (eGFR) using the CKD-EPI (Chronic Kidney Disease Epidemiology Collaboration) equation.

### Carotid–femoral pulse wave velocity measurements

The same observer performed the measurements of cfPWV values to record the pressure pulse waveform in the underlying artery [21, 22], by using a pressure tonometer (SphygmoCor system, AtCor Medical, Sydney, New South Wales, Australia). All measurements were performed in the morning with patients in supine position after a minimum of 10-min rest in a quiet, temperature-controlled room. The patients were required to abstain from smoking and consuming alcohol or coffee prior to the procedure. Records were made simultaneously with an ECG signal, which provided an *R*-timing reference. Pulse wave recordings were performed consecutively at two superficial artery sites (carotid–femoral segment). Integral software was used to process each set of pulse wave and ECG data to calculate the mean time difference between the *R*-wave and pulse wave on a beat-to-beat basis, with an average of 10 consecutive cardiac cycles. The cfPWV was calculated using the distance and mean time difference between the two recorded points. Quality indices, included in the software, were set to ensure uniformity of the data. In this study, patients with cfPWV values > 10 m/s were assigned to the high arterial stiffness group, whereas those with values ≤10 m/s constituted the low arterial stiffness group according to the ESH-ESC 2013 guidelines [10].

### Statistical analysis

Normally distributed data were expressed as mean ± standard deviation, and comparisons between patients were performed using the Student’s independent *t*-test (two-tailed). Data that were not normally distributed were expressed as median and interquartile ranges; while the Mann–Whitney U test was used for the comparisons of TG, fasting glucose, and DKK1 levels between patients. Categorical variables (expressed by numbers of patients) were analyzed by the χ^2^ test. Variables that were significantly associated with arterial stiffness in hypertensive patients were tested for independence using a forward multivariable logistic regression analysis (adapted factors: DM, age, SBP, eGFR, iPTH, and sclerostin). Since serum TG, fasting glucose, and DKK1 levels were not normally distributed; therefore, logarithmic transformations were applied to achieve normality. Correlation between clinical variables and cfPWV values in patients with hypertension was evaluated using a simple linear regression analysis, and variables that were significantly correlated with cfPWV values were tested for independence using a multivariable forward stepwise regression analysis (adopted factors: DM, age, SBP, HDL-C, BUN, creatinine, eGFR, iPTH, and sclerostin). Data were analyzed using the SPSS software for Windows (version 19.0; SPSS, Chicago, IL, USA). A *p* value < 0.05 was considered as statistically significant.

## Results

Demographic, biochemical, and clinical characteristics of the 105 hypertensive patients are shown in Table 1. The medical histories included diabetes mellitus (*n* = 46; 43.8%) and dyslipidemia (*n* = 87; 82.9%). The medications used included angiotensin-converting enzyme inhibitor (ACEI; *n* = 33; 31.4%), angiotensin receptor blocker (ARB; *n* = 60; 57.1%), β-blocker (*n* = 62; 59.0%), calcium channel blocker (CCB; *n* = 49; 46.7%), statin (*n* = 64; 61.0%), and fibrate (*n* = 17; 16.2%). Thirty-six hypertensive patients (34.3%) were included in the high arterial stiffness group. More hypertensive patients who had DM were in the high arterial stiffness group (*p* = 0.030). No statistically significant differences were observed in gender, serum DKK1 levels, ACEI, ARB, β-blocker, CCB, statin, or fibrate use between the two arterial stiffness groups. The patients in the high arterial stiffness group presented older age (*p* < 0.001), higher SBP (*p* = 0.013), serum creatinine (*p* = 0.013), iPTH (*p* = 0.003), and sclerostin levels (*p* < 0.001), and lower eGFR (*p* = 0.014) than the patients in the low arterial stiffness group.

**Table 1:** Clinical variables of the 105 hypertensive patients with or without arterial stiffness

Characteristic	All participants (*n* = 105)	Low AS group (*n* = 69)	High AS group (*n* = 36)	*p* value
Age (years)	64.51 ± 10.21	61.96 ± 10.01	69.42 ± 8.80	< 0.001*
Height (cm)	160.92 ± 8.47	161.55 ± 8.45	159.72 ± 8.50	0.296
Body weight (kg)	69.12 ± 12.31	68.71 ± 13.38	69.91 ± 10.10	0.639
Body mass index (kg/m^2^)	26.60 ± 3.61	26.16 ± 3.61	27.43 ± 3.50	0.088
cfPWV (m/s)	9.33 ± 2.76	7.78 ± 1.43	12.29 ± 2.23	< 0.001*
Systolic blood pressure (mmHg)	134.52 ± 17.85	131.41 ± 14.94	140.50 ± 21.39	0.013*
Diastolic blood pressure (mmHg)	73.72 ± 9.95	73.23 ± 9.96	74.67 ± 10.01	0.486
Total cholesterol (mg/dL)	174.51 ± 38.22	176.94 ± 36.85	169.86 ± 40.84	0.370
Triglyceride (mg/dL)	121.00 (93.50–172.00)	121.00 (94.00–174.00)	128.00 (89.50–173.50)	0.718
HDL-C (mg/dL)	45.62 ± 13.37	46.72 ± 13.86	43.50 ± 12.28	0.243
LDL-C (mg/dL)	103.25 ± 28.92	103.96 ± 26.20	101.89 ± 33.89	0.730
Fasting glucose (mg/dL)	114.00 (98.50–157.00)	116.00 (98.50–158.00)	115.00 (97.75–153.75)	0.893
Blood urea nitrogen (mg/dL)	16.91 ± 5.26	16.20 ± 4.74	18.28 ± 5.97	0.054
Creatinine (mg/dL)	1.10 ± 0.33	1.06 ± 0.28	1.19 ± 0.41	0.042*
eGFR (mL/min)	69.34 ± 20.51	72.86 ± 19.07	62.60 ± 21.72	0.014*
Total calcium (mg/dL)	9.17 ± 0.37	9.19 ± 0.37	9.14 ± 0.36	0.512
Phosphorus (mg/dL)	3.52 ± 0.51	3.56 ± 0.52	3.43 ± 0.49	0.219
iPTH (pg/mL)	50.81 ± 27.71	45.12 ± 22.04	61.72 ± 33.94	0.003*
Sclerostin (pmol/L)	69.34 ± 20.51	52.58 ± 21.66	74.77 ± 31.02	< 0.001*
Dickkopf-1 (pmol/L)	14.00 (6.06–28.82)	14.61 (5.94–30.23)	13.52 (6.02–25.51)	0.845
Female, *n* (%)	36 (34.3)	25 (36.2)	11 (30.6)	0.561
Diabetes mellitus, *n* (%)	46 (43.8)	25 (36.2)	21 (58.3)	0.030*
ACE inhibitor, *n* (%)	33 (31.4)	22 (31.9)	11 (30.6)	0.889
Angiotensin receptor blocker, *n* (%)	60 (57.1)	39 (56.5)	21 (58.3)	0.859
β-blocker, *n* (%)	62 (59.0)	37 (53.6)	25 (69.4)	0.118
Calcium channel blocker, *n* (%)	49 (46.7)	29 (42.0)	20 (55.6)	0.187
Statin, *n* (%)	64 (61.0)	46 (66.7)	18 (50.0)	0.097
Fibrate, *n* (%)	17 (16.2)	8 (11.6)	9 (25.0)	0.077

Values for continuous variables are shown as mean ± standard deviation after analysis by Student’s *t*-test; variables not normally distributed are shown as median and interquartile range after analysis by the Mann-Whitney U test; values are presented as number (%) and analysis after analysis by the chi-square test

*AS* arterial stiffness, *cfPWV* carotid–femoral pulse wave velocity, *HDL-C* high-density lipoprotein cholesterol, *LDL-C* low-density lipoprotein cholesterol, *eGFR* estimated glomerular filtration rate, *iPTH* intact parathyroid hormone, *ACE* angiotensin-converting enzyme

Patients with cfPWV values > 10 m/s were assigned to the high AS group, whereas those with values ≤10 m/s constituted the low AS group

*Values of *p* <  0.05 were considered statistically significant

Our forward multivariable logistic regression analysis, after adjusting for the factors significantly associated with arterial stiffness (diabetes mellitus, age, SBP, eGFR, iPTH, and sclerostin) showed that sclerostin (odds ratio = 1.042; 95% CI = 1.017–1.068; *p* = 0.001), age (odds ratio = 1.088, 95% CI = 1.021–1.159, *p* = 0.010), and iPTH (odds ratio = 1.025, 95% CI = 1.003–1.048, *p* = 0.026) were independent predictors of arterial stiffness in hypertensive patients (Table 2).

**Table 2:** Multivariable logistic regression analysis of the factors correlated with arterial stiffness among the 105 hypertensive patients

Variables	Odds ratio	95% confidence interval	*p* value
Sclerostin (pmol/L)	1.042	1.017–1.068	0.001*
Age	1.088	1.021–1.159	0.010*
Intact parathyroid hormone	1.025	1.003–1.048	0.026*
Systolic blood pressure	1.028	0.996–1.062	0.086
Diabetes mellitus	2.189	0.769–6.235	0.142
Estimated glomerular filtration rate	0.996	0.969–1.024	0.794

Patients with carotid–femoral pulse wave velocity values > 10 m/s were assigned to the arterial stiffness in this study

*Values of *p* <  0.05 were considered statistically significant in the forward multivariate logistic regression analysis (adopted factors: diabetes mellitus, age, systolic blood pressure, estimated glomerular filtration rate, intact parathyroid hormone, and sclerostin)

**Fig. 1:**
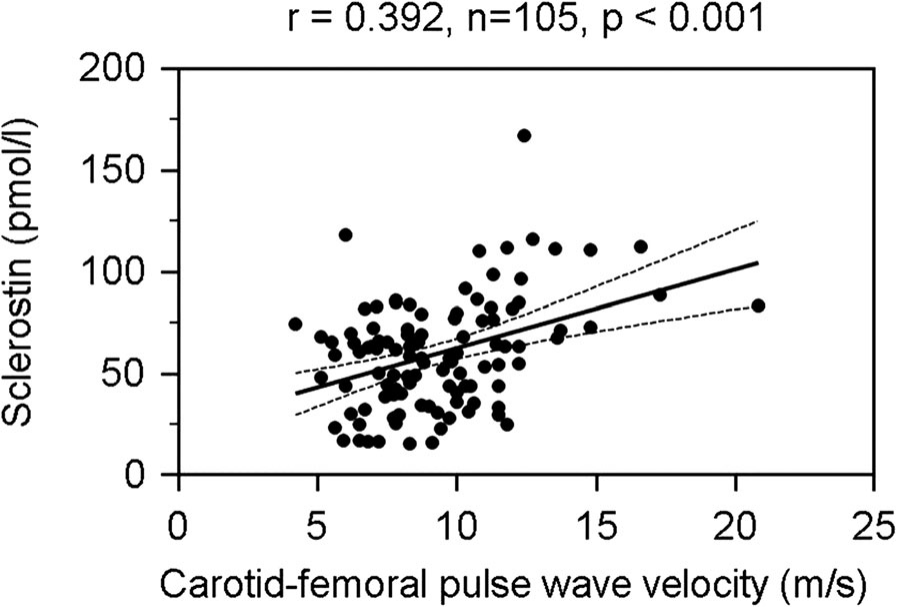
Two-dimensional scattered plots of carotid–femoral pulse wave velocity levels with serum sclerostin levels among the 105 hypertensive patients

The correlation between cfPWV values and clinical variables among patients with hypertension is shown in Table 3. Simple linear regression analysis found DM (*r* = 0.228, *p* = 0.019), age (*r* = 0.366, *p* < 0.001), SBP (*r* = 0.334, *p* = 0.001), BUN (*r* = 0.243, *p* = 0.013), creatinine (*r* = 0.369, *p* < 0.001), iPTH (*r* = 0.291, *p* = 0.003), and sclerostin levels (*r* = 0.392, *p* < 0.001) to be positively correlated with cfPWV values, whereas HDL-C (*r* = − 0.267, *p* = 0.006), eGFR (*r* = − 0.335, *p* < 0.001) was negatively correlated with cfPWV values. Log-DKK1 levels showed no significant correlation with cfPWV values. Multivariable forward stepwise linear regression analysis revealed that age (β = 0.325, adjusted R^2^ change = 0.109, *p* < 0.001), SBP (β = 0.236, adjusted R^2^ change = 0.061, *p* = 0.004), iPTH (β = 0.185, adjusted R^2^ change = 0.032, *p* = 0.022), HDL-C (β = − 0.193, adjusted R^2^ change = 0.025, *p* = 0.027), and sclerostin levels (β = 0.255, adjusted R^2^ change = 0.146, *p* = 0.003) were independent predictors of cfPWV values in our patients with hypertension. Two-dimensional scattered plots of serum sclerostin levels and cfPWV values among the 105 hypertensive patients are shown in Fig. 1.

**Table 3:** Correlation between carotid-femoral pulse wave velocity levels and clinical variables among the 105 hypertensive patients

Variables	Carotid–femoral pulse wave velocity (m/s)				
	Simple linear regression		Multivariable linear regression		
	r	*p* value	Beta	Adjusted R^2^ change	*p* value
Female	−0.154	0.116	–	–	–
Diabetes mellitus	0.228	0.019*	–	–	–
Age (years)	0.366	< 0.001*	0.325	0.109	< 0.001*
Height (cm)	− 0.017	0.866	–	–	–
Body weight (kg)	−0.003	0.975	–	–	–
Body mass index (kg/m^2^)	0.025	0.800	–	–	–
Systolic blood pressure (mmHg)	0.334	0.001*	0.236	0.061	0.004*
Diastolic blood pressure (mmHg)	0.100	0.308	–	–	–
Total cholesterol (mg/dL)	−0.110	0.263	–	–	–
Log-Triglyceride (mg/dL)	−0.001	0.991	–	–	–
HDL-C (mg/dL)	−0.267	0.006*	− 0.193	0.025	0.027*
LDL-C (mg/dL)	− 0.019	0.849	–	–	–
Log-Glucose (mg/dL)	0.001	0.998	–	–	–
Blood urea nitrogen (mg/dL)	0.243	0.013*	–	–	–
Creatinine (mg/dL)	0.369	< 0.001*	–	–	–
eGFR (mL/min)	− 0.335	< 0.001*	–	–	–
Total calcium (mg/dL)	−0.027	0.785	–	–	–
Phosphorus (mg/dL)	−0.101	0.306	–	–	–
iPTH (pg/mL)	0.293	0.003*	0.185	0.032	0.022*
Sclerostin (pmol/L)	0.392	< 0.001*	0.255	0.146	0.003*
Log-Dickkopf-1 (pmol/L)	−0.250	0.142	–	–	–

Data of triglyceride, glucose, and dickkopf-1 showed skewed distribution and therefore were log-transformed before analysis

Analysis of data was done using the simple linear regression analyses or multivariable stepwise linear regression analysis (adapted factors were diabetes mellitus, age, systolic blood pressure, HDL-C, blood urea nitrogen, creatinine, eGFR, iPTH, and sclerostin)

*HDL-C* high-density lipoprotein cholesterol, *LDL-C* low-density lipoprotein cholesterol, *eGFR* estimated glomerular filtration rate, *iPTH* intact parathyroid hormone

*Values of *p* < 0.05 were considered statistically significant

## Discussion

This study demonstrated that serum sclerostin levels were higher in hypertensive patients who had central arterial stiffness. Together with other confounders, serum sclerostin levels were also an independent marker of cfPWV values. However, serum DKK1 levels were not different among hypertensive patients by this measure of central arterial stiffness.

The aging process of blood vessel is primarily associated with changes in the arterial structure, thickening, and loss of elasticity over time, resulting in an increase in arterial stiffness [23]. Vascular calcification is associated with central arterial stiffness which leads to earlier reflected pressure waves from the arterioles toward the heart, causing an elevation in SBP and pulse pressure [7]. Patients with impaired glucose metabolism and type 2 DM were reported to have high central arterial stiffness [24]. One systematic review found that in approximately 90% of the studies, age and BP were consistently independently associated with cfPWV values, whereas DM was associated with cfPWV values in 52% of the studies [25]. A study found that BP was positively associated with the cfPWV values and was regarded as the main driver of arterial stiffening in 1052 untreated patients [26]. Similarly, we found that hypertensive patients with high arterial stiffness were older, presented with higher SBPs, and had a higher incidence of DM. Furthermore, we found older age to be the risk factors for the development of arterial stiffness after adjusting the covariates. Age and SBP were also positively associated with cfPWV values in patients with hypertension.

Central arterial stiffness transfers excessive flow pulsatility into the renal microvasculature, leading to dynamic constriction or vessel loss that contributes to lower eGFR [27]. The aortic PWV value was shown to be negatively associated with eGFR values in 2564 patients from the Chronic Renal Insufficiency Cohort (CRIC) study [28]. However, conflicting result regarding cfPWV values and eGFR was found in another clinical study [29], probably due to the differences in the number of CKD population and various disease comorbidities. Our results showed that hypertensive patients with high arterial stiffness had higher serum creatinine and lower eGFR values. HDL-C can both have anti-atheromatous effects on the arterial wall and protective effects on endothelial cell, through which may improve arterial stiffness [30, 31]. Our results showed HDL-C levels were negatively associated with cfPWV values in patients with hypertension. A similar finding was also observed in community-dwelling individuals and healthy adults in China [32, 33]. Elevation in serum parathyroid hormone (PTH) often accompanies renal impairment [34]. PTH was associated with an increased risk of nonfatal atherosclerotic disease in two prospective, community-based studies focusing on elderly patients [35]. Moreover, a recent study found that PTH had both a direct and a BP-mediated indirect effect on the cfPWV values [26]. In our study, elevated iPTH levels revealed a significant association with high arterial stiffness and iPTH levels was also positively associated with cfPWV values in patients with hypertension.

Vascular calcification is associated with arterial stiffness and osteoprogenitor cells play an important role in the bone–vascular axis which regulates calcium metabolism around the arterial wall [36, 37]. Many factors can modulate vascular calcification, such as osteoblastic differentiation, vitamin D status, matrix Gla protein or even oxidative stress [16]. However, vascular calcification physiopathology is still poorly understood [16]. One of the mechanism that affects osteoblastic differentiation and contributes to the development of vascular calcification is the Wnt/β-catenin signaling pathway [17, 18]. Sclerostin is a signaling pathway inhibitor of the Wnt/β-catenin pathway that contributes to reduced bone turnover and upregulates the calcification induced by vascular smooth muscle cells [15, 16, 38, 39]. After vascular calcification has progressed, serum sclerostin is elevated to decrease β-catenin stability in the cells and suppress the proliferation and differentiation of osteoprogenitor cells [15, 16, 37]. Clinical studies have reported that serum sclerostin levels are positively correlated with carotid intima-media thickness in hemodialysis patients, and positively associated with coronary artery or abdominal aortic calcification in non-dialysis CKD patients [40–42]. Moreover, serum sclerostin levels were found to be positively associated with brachial-ankle PWV values in kidney transplantation patients, and positively associated with cfPWV values in chronic kidney disease (CKD), hemodialysis, and postmenopausal women [20, 43–45]. In type 2 DM patients with atherosclerotic lesions, serum sclerostin level is also increased [46]. Accordingly, our study also showed that serum sclerostin levels were identified as independent predictors of central arterial stiffness in hypertensive patients. DKK1 is another inhibitor of the Wnt/β–catenin pathway [47]. However, some studies did not find the associated with arterial stiffness. Serum DKK1 levels were not found to be associated with brachial-ankle PWV values in kidney transplantation patients [20]. Another study noted plasma DKK1 levels did not associate with PWV in postmenopausal women [45]. In this study, we did not find the association between DKK1 and central arterial stiffness in hypertensive patients. Further studies are required to elucidate the relationship between central arterial stiffness and serum DKK1 level in hypertensive patients.

The limitations of our study include its cross-sectional design and the limited number of participants enrolled, which do not allow for exclusion of bias. Also, we are aware that antihypertensive medications, dyslipidemia, DM, and lifestyle modifications may also affect cfPWV values. In addition, we did not assess vascular endothelial function through flow-mediated dilatation or inflammation markers such as C-reactive protein and vitamin D status.

Despite contradictory findings that showed serum sclerostin levels to be positively correlated with carotid intima-media thickness and inversely correlated with cfPWV values in 67 subjects at Catania, Italy [48], it may be explained by the differences in ethnicity of populations, inclusion or exclusion criteria, race, or locations between these two studies. Additional long-term prospective studies or randomized controlled trials are needed to confirm our finding that sclerostin levels can predict central arterial stiffness among hypertensive patients.

## Conclusions

For the inhibitors of the Wnt/β-catenin pathway, serum sclerostin levels, not DKK1, are positively correlated with cfPWV values in patients with hypertension. Meanwhile, serum sclerostin levels were found to be independent risk factors for the development of arterial stiffness in our patients.

## Abbreviations

ACEi: Angiotensin-converting enzyme inhibitors
ARB: Angiotensin receptor blockers
BP: Blood pressures
BUN: Blood urea nitrogen
CCB: Calcium channel blockers
cfPWV: Carotid–femoral pulse wave velocity
CI: Confidence interval
CKD: Chronic kidney disease
CRIC: Chronic renal insufficiency cohort
DBP: Diastolic blood pressure
DKK1: Dickkopf-1
DM: Diabetes mellitus
eGFR: Estimated glomerular filtration rate
HDL-C: High-density lipoprotein cholesterol
iPTH: Intact parathyroid hormone
LDL-C: Low-density lipoprotein cholesterol
LRP5/6: Low-density lipoprotein receptor-related protein
PWV: Pulse wave velocity
SBP: Systolic blood pressure
TCH: Total cholesterol
TG: Triglycerides
